# Analyses of publicly available genomics resources define FGF-2-expressing bladder carcinomas as EMT-prone, proliferative tumors with low mutation rates and high expression of CTLA-4, PD-1 and PD-L1

**DOI:** 10.1038/sigtrans.2016.45

**Published:** 2017-03-17

**Authors:** Elizabeth A McNiel, Philip N Tsichlis

**Affiliations:** 1 Molecular Oncology Research Institute, Tufts Medical Center, Boston, MA, USA; 2 Cummings School of Veterinary Medicine, Tufts University, North Grafton, MA, USA

## Abstract

Fibroblast growth factor 2 (FGF-2) is overexpressed in a subset of invasive bladder carcinomas and its overexpression correlates with poor prognosis. Analyses of publicly available databases addressing the molecular mechanisms that may be responsible for the poor prognosis of these tumors, revealed that FGF-2 expression correlates positively with the expression of epithelial to mesenchymal transition (EMT)-promoting transcription factors and with changes in gene expression that are characteristic of EMT. The same analyses also revealed that FGF-2 correlates negatively with the expression, mutation and copy number variations of FGFR-3, all of which are associated with noninvasive bladder carcinomas. Finally, they showed that FGF-2 expression correlates with the expression of FGFR-1, the expression of the IIIc variant of FGFR-2 and with the expression of Akt3. The latter observation is significant because our earlier studies had shown that Akt3 regulates FGFR-2 alternative splicing, shifting the balance toward the IIIc relative to the IIIb FGFR-2 splice variant. As the IIIc variant is recognized by FGF-2, while the IIIb variant is not, we conclude that Akt3 may facilitate the FGF-2 response. FGF-2 is known to promote the expression of KDM2B, which functions in concert with EZH2 to repress the EZH2-targeting microRNA miR-101, activating a switch, which stably upregulates EZH2. The cancer genome atlas (TCGA) data showing a correlation between KDM2B and EZH2 expression and Oncomine data, showing a correlation between KDM2B and tumor progression, strongly support the role of the FGF-2/KDM2B/miR-101/EZH2 pathway in bladder cancer. These observations combined, suggest a model according to which FGF-2 induces EMT, cell proliferation and cancer stem cell self-renewal by coupling the Akt3 and KDM2B-controlled pathways outlined above, in bladder carcinomas. Further analyses of publicly available databases, revealed that FGF-2-expressing bladder carcinomas carry fewer genetic alterations and they tend to express high levels of CTLA-4, PD-1 and PD-L1, which suggests immune blockade by checkpoint activation. EMT, enhanced proliferation and immune checkpoint activation combined, may be responsible for the poor prognosis of FGF-2-expressing bladder carcinomas.

## Introduction

Bladder carcinomas are classified as either superficial or invasive, with the two types of tumors characterized by different sets of genomic alterations.^1,2^ Although occasionally superficial tumors evolve to become invasive, the two types of bladder cancer represent distinct biological entities.^1,2^ It is interesting that although activating mutations in *FGFR* genes (primarily *FGFR-3*) are common in the superficial form of bladder cancer, overexpression of fibroblast growth factor 2 (FGF-2) is often observed in invasive bladder cancer.^3–5^

Our prior studies suggested at least two molecular mechanisms that may contribute to the invasiveness of FGF-2-expressing bladder carcinomas. The first study linked FGF-2 with Akt3 through the alternative splicing of *FGFR-2.* ^6^ The receptor encoded by this gene contains three immunoglobulin-like domains in its extracellular ligand-binding region. The second half of the third domain is encoded by either exon 8, or exon 9 and the predominant FGFR-2 isoform is determined by shifts in the alternative splicing between these two mutually exclusive exons.^7,8^ The exon 8-containing isoform, which is known as the IIIb isoform, does not recognize FGF-2 and is expressed in epithelial cells. The exon 9-containing isoform, which is known as the IIIc isoform, recognizes FGF-2 and is expressed in mesenchymal cells. Moreover, the expression of the IIIc isoform in various types of human cancer has been linked to epithelial to mesenchymal transition (EMT) and increased invasiveness, metastatic potential and cancer stem cell self-renewal.^9,10^ Our studies have shown that Akt3, and secondarily Akt1, transduce signals that shift the alternative splicing of FGFR-2 toward the IIIc isoform, which is recognized by FGF-2. Specifically, we have shown that Akt3 phosphorylates IWS1, a scaffold protein involved in the assembly of a transcriptional elongation complex on the Ser2-phosphorylated C-terminal domain of RNA Pol II and that the complex assembled by phosphorylated IWS1 gives rise to epigenetic changes that shift the splicing of FGFR-2 toward the IIIc isoform.^6^

The second study revealed that FGF-2 activates DYRK1A, which phosphorylates CREB and induces the expression of the histone H3K36me2/me1 demethylase KDM2B. Following its induction, KDM2B functions in concert with EZH2 to regulate cell proliferation, migration, angiogenesis, transformation and stem and progenitor cell maintenance and self-renewal in human tumors, including bladder cancer.^11^ Its role in stem and progenitor cell biology depends on its function as a master regulator of a set of microRNAs which target several components of the polycomb complexes.^12^ The FGF-2-induced KDM2B, in concert with pre-existing EZH2, represses the expression of these microRNAs, leading to the upregulation of BMI1, EZH2, SUZ12 and RING1B and the functional activation of the polycomb complexes PRC1 and PRC2. One of the microRNAs targeted by this pathway is miR-101. As miR-101 targets EZH2, its repression establishes a feed-forward loop, which results in the stable upregulation of EZH2 and the stable downregulation of miR-101.^11,12^

To address the potential contribution of these pathways to the biology of FGF-2-expressing bladder carcinomas, we searched publicly available genomics resources of invasive bladder cancer. The focus of the search was the relationship between the expression of FGF-2 and regulators and markers of EMT, between the expression of FGF-2, Akt3 and the FGFR-2 splicing variants, and between FGF-2/KDM2B and EZH2. The results support a model according to which FGF-2 promotes EMT, cell proliferation and cancer stem cell self-renewal, by coupling the Akt3-controlled^6^ and the FGF-2/KDM2B/miR-101/EZH2-controlled pathways.^11,12^

The preceding data identify FGF-2-expressing bladder carcinomas as a tumor subtype that is prone to EMT and is characterized by an invasive phenotype and poor prognosis. Viewed in the context of the results of our earlier studies, these data suggest that the tumor phenotype may be due to the promotion of FGF-2 responsiveness by the Akt3/IWS1 pathway and the induction of EMT and stem cell self-renewal by FGF-2 via the FGF-2/KDM2B/miR-101/EZH2 pathway. Further searches of the same genomic resources revealed that high FGF-2 tumors are also characterized by lower numbers of mutations per megabase and gene copy number variations, as well as by high expression of immune checkpoint genes including CTLA-4, PDCD1 (PD-1) and CD274 (PD-L1).^13–18^ These data suggest that tumors with high FGF-2-expression also tend to exhibit immune checkpoint activation, which may contribute, along with EMT and enhanced proliferation, to the poor prognosis of FGF-2-expressing bladder carcinomas. Immune checkpoint activation is required for the response to checkpoint inhibitors. In contrast, low mutational load in tumors has been linked to resistance to immune checkpoint inhibition. Our observations raise the question whether FGF-2 expression identifies a subset of tumors with low numbers of genetic alterations that may respond to immune checkpoint inhibitors.

## Materials and methods

### Data sets

The cancer genome atlas data (TCGA) project for invasive bladder cancer currently includes 412 primary tumors and the data are publicly available for download at https://gdc-portal.nci.nih.gov/projects/TCGA-BLCA. These tumors have been analyzed using high-throughput molecular technologies including gene (*N*=408) and miRNA expression (*N*=409), copy number variations and single-nucleotide variants (*N*=410) and whole-exome sequencing. A detailed analysis of a set of 131 invasive tumors was previously published and included the classification of tumors into various categories based on mutational landscape and on other features including mRNA expression.^19^

As the TCGA data set focuses on invasive primary bladder cancers, we evaluated additional data sets that included normal tissues, superficial bladder carcinomas, as well as invasive tumors. Microarray-based gene expression data sets for bladder cancer used in these analyses, were limited to those reporting the expression of genes of interest, including those encoding *KDM2B* and *EZH2*. This included Lindgren *et al.,* ^20^ an expression set comprising 75 bladder cancers characterized by grade, TP53 and FGFR-3 mutation, and Lee *et al.,* ^21^ which comprised 256 bladder cancers of various pathologic type and stages.

### Analysis tools

Readily available analysis software was used to query bladder cancer data sets. This included Oncomine Research Addition Software (oncomine.com),^22^ the University of California at Santa Cruz Xena Browser (xena.ucsc.edu/)^23^ and Memorial Sloan Kettering’s cBioPortal (cbioprotal.org).^24,25^

### Survival analysis

The relationship between FGF-2 and survival of patients with invasive bladder cancer in the TCGA database was investigated using the Xena browser. Primary tumors with gene expression data were selected to generate Kaplan–Meier curves and calculate log-rank statistics.^23^

### Expression clusters

We used the previously published expression cluster analysis,^19^ which places TCGA invasive bladder cancers (*N*=129) into four distinct categories. Using cBioPortal, we determined the FGF-2 expression level of these tumors. FGF-2 expression was compared across categories using a Kruskal–Wallis test.

### Genomic alterations

To evaluate for associations between FGF-2 and mutational status of invasive bladder cancers, FGF-2 expression levels were downloaded from cBioPortal for 130 tumors with complete mutation data. Tumors with FGF-2 expression levels greater than the 75th percentile were defined as having high FGF-2 expression, whereas the tumors that fell below the 75th percentile were defined as low FGF-2-expressing tumors. Next, we addressed the distribution of genetic alterations in FGF-2 high and low groups focusing on (i) mutations per megabase and (ii) copy number variations per tumor both obtained from cBioPortal. Statistical comparisons between FGF-2 expression categories with respect to (i) mutations per megabase and (ii) copy number variations per tumor were performed using a Student’s *t*-test.

### Correlations

The TCGA data set was used to explore relationships between gene/exon expression, non-silent genetic mutations, and/or copy number alterations. Figures were generated using the visualization tools of the Xena browser.^23^ Expression data were downloaded from cBioPortal for the calculation of Spearman and Pearson correlation coefficients and associated *P*-values.

## Results

### FGF-2 expression is associated with EMT and poor prognosis in primary human bladder carcinomas

**Figure 1:**
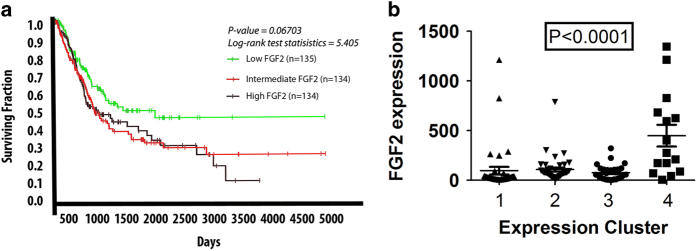
Bladder carcinomas expressing higher levels of FGF-2 exhibit worse prognosis. (**a**) Kaplan–Meier survival curve for 407 invasive bladder carcinoma patients, stratified for the expression of FGF-2. Primary data from the TCGA database processed using the UCSC Xena browser. Expression was based on RNA-Seq data. (**b**) Hierarchical clustering of 129 invasive bladder carcinomas from the TCGA data set demonstrates that high FGF-2-expressing tumors belong primarily to subtype IV of invasive bladder carcinomas. FGF-2, fibroblast growth factor 2; TCGA, the cancer genome atlas.^19^

The expression of FGF-2 in human bladder cancer has been linked to aggressive behavior and poor prognosis.^3,5,26,27^ Using the UCSC Xena Genome Browser, we generated Kaplan–Meier survival curves for tumors based on FGF-2 expression, which confirmed this finding in a provisional set of 407 primary muscle invasive bladder carcinomas in the cancer genome atlas (TCGA) database (Figure 1a). Hierarchical clustering of the gene expression profiles of a subset of these tumors (*n*=129), revealed that they can be classified into four distinct subgroups.^19^ Addressing the distribution of FGF-2 expression among the four subgroups, revealed that although high FGF-2-expressing tumors can be found in all four, only subgroup IV contains a high percentage of such tumors (Figure 1b). Tumors that belong to this subgroup tend to have non-papillary morphology and they express low levels of luminal epithelial markers and low levels of microRNAs of the miR-200 family.^19^

**Figure 2:**
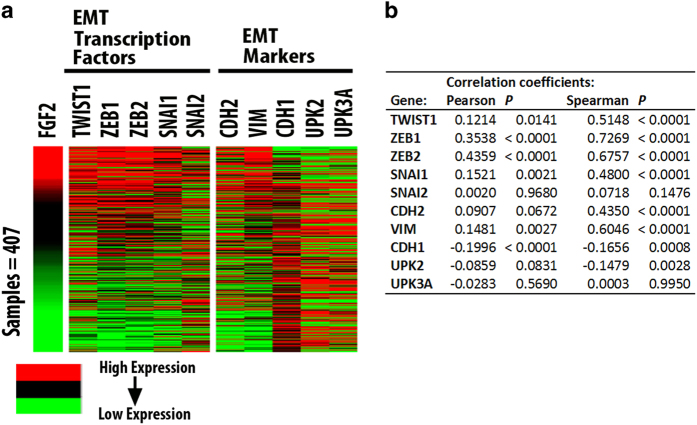
FGF-2 expression in bladder carcinomas correlates with the expression of EMT-promoting transcription factors and the expression of EMT markers. (**a**) Heat maps showing gene expression in a set of 407 invasive bladder carcinomas from the TCGA database. The heat maps examine the correspondence between the expression of FGF-2 and EMT-promoting transcription factors, and the correspondence between the expression of FGF-2 and the mesenchymal markers N-Cadherin (CDH2) and vimentin (VIM) and epithelial markers E-Cadherin (CDH1), Uroplakin II (UPK2) and IIIa (UPK3A). Gene expression was determined from the analysis of RNA-Seq data. (**b**) Correlation coefficients for expression of genes depicted in heat maps are provided in tabular form. EMT, epithelial to mesenchymal transition; FGF-2, fibroblast growth factor 2; TCGA, the cancer genome atlas.

To address the mechanism by which FGF-2 promotes the aggressiveness of invasive bladder carcinomas, we examined the gene expression profiles of 407 muscle invasive bladder carcinomas in the TCGA database and we observed that FGF-2 expression correlates with the expression of transcription factors that promote epithelial to mesenchymal transition (EMT) and stemness, as well as with the expression of mesenchymal markers, such as vimentin (VIM), and N-Cadherin (CDH2; Figure 2). This finding suggests a molecular link between FGF-2 and EMT.

### The expression of FGF-2 exhibits a negative correlation, with the expression, mutation and copy number variations of the gene encoding FGFR-3

**Figure 3:**
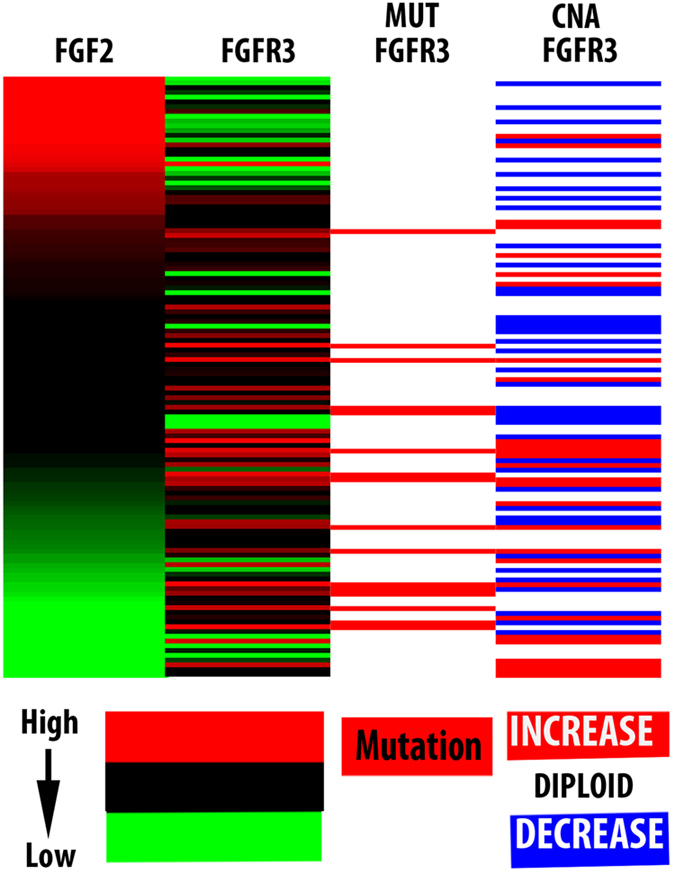
FGF-2 expression exhibits a negative correlation with the expression, mutation and copy number variations of FGFR-3 in human bladder carcinomas. Heat maps were generated by analyzing the gene expression profiles of 129 bladder carcinomas from the TCGA database with curated mutation data. The analyses and heatmap generation were produced using the UCSC XENA browser. FGF-2, fibroblast growth factor 2; TCGA, the cancer genome atlas.

Bladder carcinomas frequently overexpress, or carry mutations and/or copy number variations in *FGFR* genes (primarily *FGFR-3*).^2^ However, genetic changes in *FGFR-3* are more common in superficial, rather than invasive bladder carcinomas,^2^ which may overexpress FGF-2. This suggests that the tumors that express high levels of FGF-2 and the tumors that overexpress FGFR-3, or carry a genetically altered *FGFR-3* gene may not overlap, or they may overlap only partially. To address this question, we examined the correlation between FGF-2 and FGFR-3 expression or FGF-2 expression and FGFR-3 genetic alterations in a set of 129 bladder carcinomas in the TCGA database for which curated sequence data were available. The results confirmed the prediction by revealing negative correlations, almost mutual exclusivity, between FGF-2 and FGFR-3 expression or between FGF-2 expression and FGFR-3 mutation or amplification (Figure 3). We conclude that the overexpression of FGF-2 and the overexpression or genetic alteration of FGFR-3 occur in minimally overlapping tumor sets.

### FGF-2 expression in bladder carcinomas correlates with the expression of Akt3 and with a shift in the alternative splicing of FGFR-2 toward the EMT-promoting IIIc isoform

**Figure 4:**
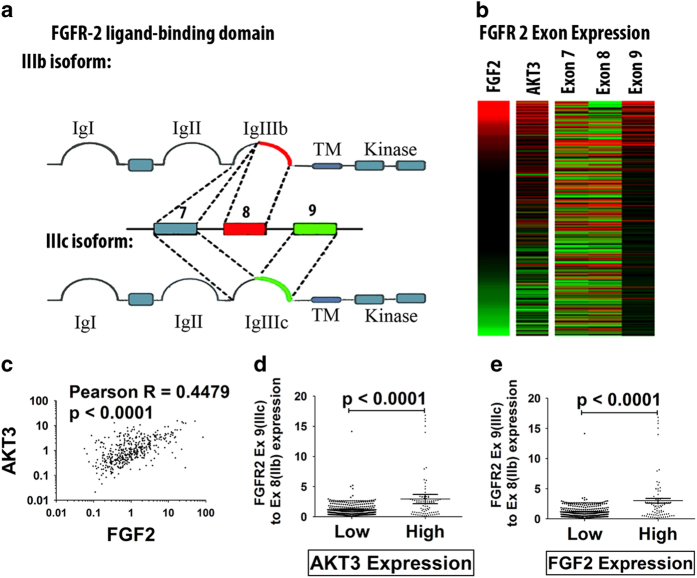
FGF-2 expression in invasive bladder carcinomas in the TCGA database correlates with the expression of Akt3 and with a shift in the splicing of FGFR-2 toward the Exon 9-containing IIIc isoform. (**a**) Alternative splicing of FGFR-2. The extracellular domain of FGFR-2 contains three Ig-like loops. The third loop is encoded by exon 7 and the mutually exclusive exons 8 and 9. Although the inclusion of exon 8 gives rise to the IIIb isoform, its exclusion gives rise to the IIIc isoform. The IIIb isoform is expressed in epithelial cells and it is not recognized by FGF-2, whereas the IIIc isoform is expressed in mesenchymal cells and invasive and metastatic cancer cells and it is recognized by FGF-2. (**b**) Heatmap showing the expression of FGF-2, Akt3 and exons 7, 8 and 9 of FGFR-2 in 407 invasive bladder carcinomas in the TCGA data set. (**c**) Correlation between the expression of Akt3 and FGF-2 in the same tumors. (**d**) Ratio of expression of FGFR-2 IIIc to IIIb in invasive bladder carcinomas expressing high and low levels of Akt3. (**e**) Ratio of expression of FGFR-2 IIIc to IIIb in invasive bladder carcinomas expressing high and low levels of FGF-2. FGF-2, fibroblast growth factor 2; TCGA, the cancer genome atlas.

Further analyses of 407 carcinomas in the TCGA data set revealed that tumors expressing high levels of FGF-2 tend to also express high levels of Akt3. Moreover, FGF-2 and Akt3 expression were found to correlate with the expression of the exon 9-containing IIIc isoform of FGFR-2, rather than the exon 8-containing IIIb isoform (Figure 4). Given our earlier studies showing that Akt3 shifts the splicing of FGFR-2 toward the IIIc isoform by phosphorylating IWS1,^6^ this finding suggests that the expression of Akt3 and the expression of the IIIc isoform of FGFR-2 may be causally linked. Moreover, given that FGF-2 signals via the IIIc isoform of FGFR-2, which is normally expressed in mesenchymal cells and in invasive and metastatic tumors undergoing EMT,^9,10^ these findings also suggest that the EMT-promoting activity of FGF-2 is under the control of Akt3 which promotes the alternative splicing of FGFR-2 by phosphorylating IWS1.

### The FGF-2/KDM2B-EZH2/miR-101/EZH2 pathway in bladder cancer

**Figure 5:**
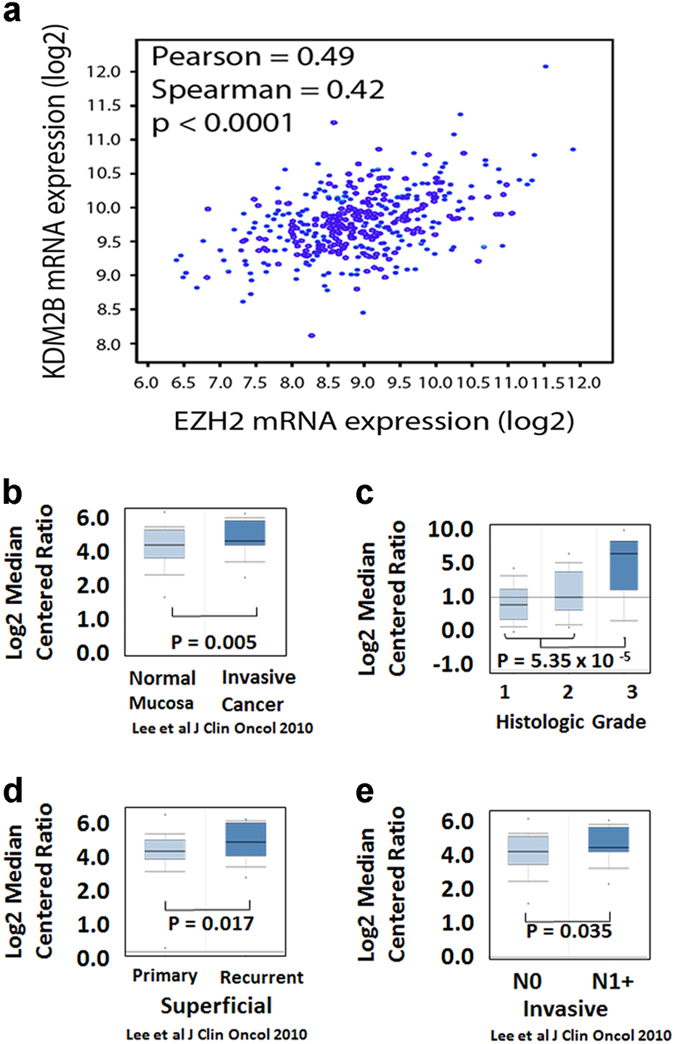
Expression of the histone demethylase KDM2B, which is upregulated by FGF-2, is associated with an invasive bladder cancer phenotype. (**a**) The expression of KDM2B correlates with the expression of EZH2 in invasive bladder carcinomas in the TCGA data set. (**b**–**e**). Correlation of gene expression with the transformation, invasiveness and metastatic potential of human urothelium. Gene expression data were extracted from publicly available data sets and they were analyzed with Oncomine. (**b**) KDM2B is expressed at higher levels in urothelial cancer, than in normal mucosa. (**c**) KDM2B is expressed at higher levels in tumors with high histologic grade. (**d**) KDM2B is expressed at higher levels in recurrent superficial cancers. (**e**) KDM2B is expressed at higher levels in invasive tumors with lymph node metastases. FGF-2, fibroblast growth factor 2; TCGA, the cancer genome atlas.

We had shown earlier that FGF-2 induces the expression of KDM2B, which functions in concert with EZH2 to repress miR-101 and to upregulate the miR-101 target EZH2. In addition, we had shown that the upregulation of KDM2B by FGF-2 results in a stable switch from a state of high miR-101 and low EZH2 expression to a state of low miR-101 and high EZH2 expression.^11^ The importance of this pathway in bladder cancer was suggested from the analysis of a small number of bladder carcinoma samples, which revealed a correlation between the expression of FGF-2 and KDM2B, FGF-2 and EZH2, as well as EZH2 and KDM2B.^11^ Gene expression correlations in the set of 407 invasive bladder carcinomas in the TCGA database revealed an excellent correlation between KDM2B and EZH2, providing additional evidence for the activation of this pathway in bladder cancer (Figure 5a).

### Activation of the FGF-2/KDM2B-EZH2/miR-101/EZH2 pathway is associated with bladder cancer progression

Our earlier studies had shown that the activation of the FGF-2/KDM2B-EZH2/miR-101/EZH2 pathway in bladder and other cancers, promotes cell migration, cancer stem cell self-renewal, cell proliferation and angiogenesis.^11,12^ Cell migration is a function often associated with EMT, which is known to be induced by EZH2, one of the effectors of this pathway.^28,29^ We therefore suggest that the correlation between FGF-2 and EMT in bladder cancer (Figure 2) may be due to the induction of EMT via the FGF-2/KDM2B-EZH2/miR-101/EZH2 pathway, which is active in these tumors. The induction of angiogenesis by FGF-2 may also be mediated by the same pathway, via EZH2, a known promoter of angiogenesis.^11^ Finally, FGF-2 may also promote stem cell self-renewal and differentiation via the same pathway. This proposition is based in our earlier observations showing that KDM2B, in concert with EZH2, represses a set of microRNAs that regulate several members of the polycomb complexes, including EZH2, SUZ12, BMI1 and RING1B.^12^ We recognize that these observations were made in studies focusing on breast rather than bladder cancer. However, we believe that these observations identify basic mechanisms that may contribute to the phenotype of multiple cancer types, including bladder cancer. Moreover, gene expression studies support common molecular subtypes (luminal and basal) for both breast and bladder cancer.^30,31^

Cell proliferation is also promoted by both KDM2B and EZH2 in tumors with an activated FGF-2/KDM2B-EZH2/miR-101/EZH2 pathway. First, EZH2 and KDM2B are known to induce the expression of cyclin D1 and to promote the progression from the G1 to the S phase of the cell cycle.^12,32^ Second, global gene expression profiling has shown that the expression of both KDM2B and EZH2 is associated with changes in the expression of many regulators of the cell cycle.^33–35^ Finally, gene expression correlations in the TCGA database, revealed significant correlations between KDM2B and several cell cycle regulators (Table 1).

Consistent with the proposed role of the FGF-2/KDM2B-EZH2/miR-101/EZH2 pathway in bladder cancer,^11^ were data we obtained by analyzing publicly available data sets in Oncomine.^20–22^ These data show that KDM2B is expressed at higher levels in bladder cancer than in the normal urothelium (Figure 5b). In superficial bladder carcinomas expressing KDM2B, the expression is higher in high-grade and recurrent tumors, which are at high risk of progression to muscle invasive carcinomas, than in non-recurrent or low-grade tumors (Figures 5c and d). Finally, among invasive carcinomas, KDM2B expression is higher in lymph node metastases, rather than in the primary tumor (Figure 5e).

### The phenotype of bladder carcinomas expressing high levels of FGF-2 depends on the coupling of two epigenetically controlled pathways; a model

**Figure 6:**
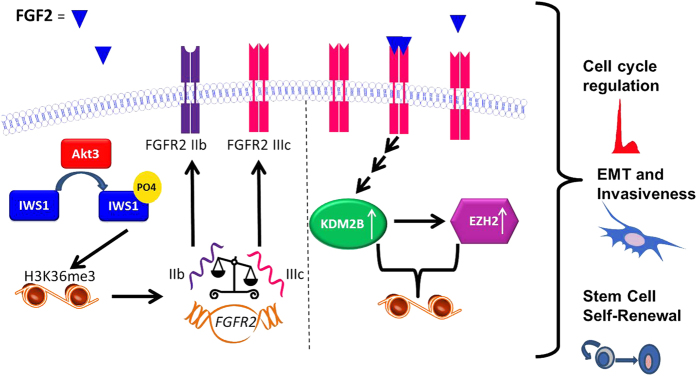
FGF-2 promotes the malignant progression of invasive bladder carcinomas by coupling two epigenetically controlled pathways: a model. FGF-2 induces the expression of KDM2B and EZH2. The Akt3 kinase, whose expression correlates with the expression of FGF-2 and may be regulated by this growth factor, promotes a shift in FGFR-2 alternative splicing toward the FGFR-2 IIIc splice form, which is recognized by FGF-2, thus enhancing the FGF-2 response. Finally, FGF-2 promotes EMT, cancer stem cell self-renewal and cell cycle progression. The mechanisms of KDM2B/EZH2 regulation by FGF-2 and the mechanism by which Akt3 regulates the alternative splicing of FGFR-2 have been published.^6,11^ The regulation of cell migration and invasiveness and the regulation of cancer stem cell self-renewal by KDM2B have also been described previously.^11,12^ Finally, the regulation of the cell cycle by KDM2B has been observed earlier, and it is supported by gene expression data in prior literature.^33,55^ EMT, epithelial to mesenchymal transition; FGF-2, fibroblast growth factor 2.

Data derived from the analyses of publicly available databases, which were described in the preceding paragraphs, revealed that FGF-2 expression correlates with the expression of Akt3 and with a shift of the alternative splicing of FGFR-2 toward the IIIc isoform (Figure 4). Given that Akt3 is known to promote FGFR-2 alternative splicing toward the IIIc isoform, we propose that tumors expressing high levels of FGF-2 upregulate Akt3 via an unknown mechanism and that Akt3 phosphorylates IWS1, initiating a pathway that tips the balance between FGFR-2 IIIb and IIIc toward IIIc (Figure 6). FGF-2 and Akt3 expression also correlate with the expression of EMT-promoting transcription factors and EMT markers (Figure 2). This observation is again consistent with the preceding data because the IIIc isoform of FGFR-2, but not the IIIb isoform, is recognized by FGF-2 and it has been linked to EMT.^9,10^ A mechanism by which FGF-2 and the IIIc isoform of FGFR-2 promote EMT, stem cell self-renewal and cell proliferation was suggested by our earlier studies showing that FGF-2 induces the expression of KDM2B.^11,12^ Following its induction, EZH2 promotes EMT.^28,29^ In addition, KDM2B in concert with EZH2, regulates cancer stem cell self-renewal by repressing several microRNAs, which target BMI1 and RING1B (PRC1), as well as EZH2 and SUZ12 (PRC2).^12^ Finally, KDM2B and EZH2, regulate cyclin D1 and other cell cycle regulators (Table 1), promoting cell cycle progression.^12,32,33,35^ Overall, these data suggest that the invasiveness and poor prognosis of bladder carcinomas expressing high levels of FGF-2 is due to the coupling of two epigenetically controlled pathways, one that is activated by FGF-2 signaling and another one that regulates the responsiveness to FGF-2.

### Bladder carcinomas expressing high levels of FGF-2 are characterized by low mutation rates and elevated expression of CTLA-4, PD-1 and PD-L1

**Figure 7:**
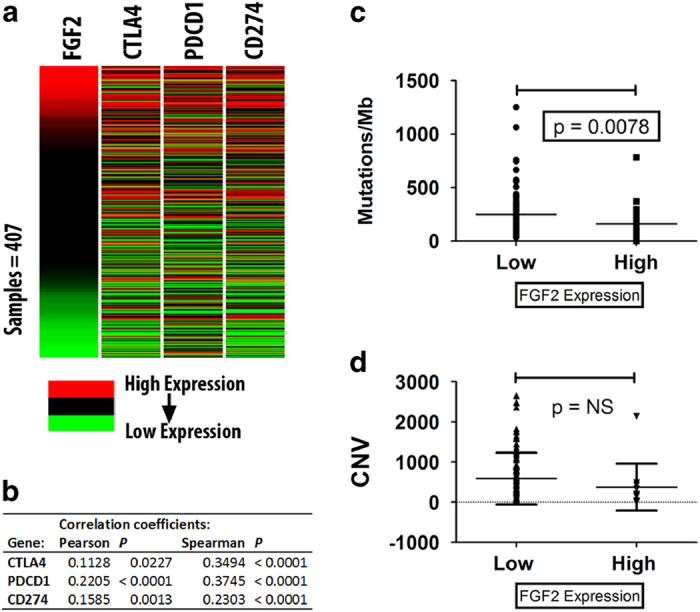
FGF-2-expressing bladder carcinomas express high levels of CTLA-4, PD-1 and PD-L1 and harbor fewer mutations and copy number gene variations. (**a**, **b**) Heat maps and correlation coefficients between the expression of FGF-2 and CTLA-4, PDCD1 (PD-1) and CD274 (PD-L1). (**c**, **d**) Invasive bladder carcinomas expressing high levels of FGF-2 harbor fewer mutations and copy number variations than invasive bladder carcinomas expressing low levels of FGF-2. FGF-2, fibroblast growth factor 2; TCGA, the cancer genome atlas.

Growing tumors utilize multiple strategies to block their elimination by the host immune system. One of these strategies is the activation of immune checkpoints that control feedback loops that inhibit the antitumor immune response.^13–18^ Activation of immune checkpoints is tumor-protective and tends to be associated with poor prognosis. Given that high FGF-2 is an indicator of poor prognosis in bladder cancer, we asked whether FGF-2 expression may also be associated with immune checkpoint activation. The results in Figure 7a confirmed that the expression of FGF-2 indeed correlates with the expression of CTLA-4, PDCD1 (PD-1) and CD274 (PD-L1) in a set of 407 invasive bladder carcinomas from the TCGA database. This observation suggests that FGF-2-expressing tumors indeed block the antitumor immune response via checkpoint activation, and that this may be partially responsible for the poor prognosis of tumors overexpressing FGF-2.

Although immune checkpoint activation is associated with bad prognosis, such tumors are also more likely to respond to immune checkpoint inhibitors.^13^ As it has been reported that low mutation rates in various types of human cancer, including bladder carcinomas, are associated with diminished responsiveness to such inhibitors,^13,36,37^ we examined the TCGA data on bladder cancer to determine the frequency of genetic alterations in tumors expressing high or low levels of FGF-2. The results (Figures 7b and c) revealed that high FGF-2 correlates with low frequency of genetic changes. We interpret this unexpected finding to suggest that there may be two distinct sets of tumors with low frequency of genetic alterations, one low FGF-2 set with no checkpoint activation, which is resistant to checkpoint inhibition, and another set with high FGF-2 and checkpoint activation, which may be sensitive to checkpoint inhibition.

## Discussion

Data presented in this report show that FGF-2-expressing tumors represent a subset of tumors characterized by Akt3 overexpression, alternative splicing of FGFR-2 toward the IIIc splice variant, which facilitates the response to FGF-2,^6^ and activation of the FGF-2/KDM2B-EZH2/miR-101/EZH2 pathway.^11,12^ FGF-2-expressing tumors also tend to express high levels of CTLA-4, PD-1 and PD-L1, which suggests immune checkpoint activation as a mechanism for the blockade of the host immune response against the tumor. The same tumors harbor genetic alterations at low frequency, which has been linked to diminished responsiveness to immune checkpoint inhibitors.^13,36,37^ These findings combined, provide a mechanistic explanation for the poor prognosis of the FGF-2-expressing tumors. However, they also raise a question that needs to be addressed. The correlation of FGF-2 expression with biomarkers of both sensitivity and resistance to immune checkpoint inhibition, suggests that there may be two distinct sets of tumors with low frequency of genetic alterations, one with high FGF-2 expression and immune checkpoint activation, which tend to be sensitive to the treatment and another one with low FGF-2 expression and no immune checkpoint activation, which tend to be resistant.

KDM2B and EZH2 are members of multiple epigenetic complexes. EZH2 is the catalytic component of PRC2 (ref. 38) and KDM2B is a member of a variant PRC1 complex that binds GC rich DNA regions and represses transcription.^39^ Quantitative proteomic studies have also shown that KDM2B and EZH2 are members of other complexes.^40^ Some of the proteins in these complexes, such as KDM6A, are encoded by genes that are frequently mutated in invasive bladder cancer.^19,41,42^ Moreover, some bladder cancer mutations inactivate enzymes, which erase histone marks that are catalyzed by KDM2B or EZH2. For example KDM6A, which is inactivated in 24% of invasive bladder carcinoma,^19^ normally erases H3K27me3 marks induced by EZH2.^43^ These links provide support to the hypothesis that KDM2B and EZH2 have an important role in the biology of invasive bladder cancer. Exploring these links may help us understand the molecular mechanisms by which KDM2B, EZH2 and genetic alterations associated with bladder cancer may contribute to the oncogenic transformation of the human urothelia.

Given the importance of the FGF-2/KDM2B-EZH2/miR-101/EZH2 axis in bladder cancer, we hypothesize that there will be clinical utility to the therapeutic targeting of this pathway. Although inhibitors of EZH2 are in clinical development,^44,45^ our preliminary data suggest that treatment with the EZH2 inhibitors as single agents may be ineffective. (i) We have shown that the knockdown of KDM2B is only partially rescued by the overexpression of EZH2, suggesting that KDM2B functions not only by upregulating EZH2, but also by promoting additional pro-oncogenic activities;^12^ (ii) although the knockdown of EZH2 has profound effects on the viability and the proliferation of human and canine bladder cancer cell lines with few cells surviving beyond 3 days after selection, the robust inhibition of its histone methyltransferase activity by GSK343 (ref. 46) does not (Foltopoulou and McNiel Data not shown). This suggests that the enzymatic inhibition of EZH2, in the absence of its downregulation, is not sufficient to elicit a strong biological response. This tentative conclusion is further supported by the observation that treatment of the same cells with DZNep (3-Deazaneplanocin A), which not only inhibits the enzymatic activity of EZH2, but also induces its degradation,^47–49^ results in robust inhibition of cell proliferation. Overall, these data also suggest that targeting EZH2 in these tumors, without targeting KDM2B may not be sufficient to inhibit tumor growth.

The preceding data suggest that inhibition of KDM2B, which would downregulate EZH2 expression, may act synergistically with the inhibition of EZH2 by GSK343 and other inhibitors of the enzymatic activity of EZH2. As selective inhibitors of KDM2B are not currently available, we can only block it indirectly by inhibiting its induction by growth factors, such as FGF-2. Our data to date revealed that inhibition of DYRK1A was effective in blocking the induction of KDM2B by FGF-2. Inhibitors of AKT, MEK, PKA, PKC and CAMKII were ineffective.^11^ On the basis of these considerations, it would be informative to address whether urothelial carcinomas with an active FGF-2/KDM2B-EZH2/miR-101/EZH2 axis, are sensitive to catalytic inhibitors of EZH2, in combination with inhibitors of the induction of KDM2B by FGF-2 or VEGF. The latter include inhibitors of DYRK1A,^50^ FGFR inhibitors^51^ and inhibitors of CBP/p300, a co-factor of CREB,^52–54^ which is a downstream effector of DYRK1a.^11^ Alternatively, miR −101 may also be effective because it will downregulate EZH2 and may also indirectly downregulate KDM2B.^11^

In summary, in this report we attempted an integration of data from publicly available databases, with our own experimental data, to explain the invasiveness and poor prognosis of FGF-2-expressing bladder carcinomas. Based on the results of this integration, we proposed therapeutic strategies that may be effective in targeting these tumors.

## Figures and Tables

**Table 1:** Correlation between cell cycle regulatory genes and KDM2B

*Cell cycle phase: G1/S*			*S*			*G2/M*			*M*		
*Gene*	*Pearson*	*Spearman*	*Gene*	*Pearson*	*Spearman*	*Gene*	*Pearson*	*Spearman*	*Gene*	*Pearson*	*Spearman*
*CDKN1A*	−0.27	−0.3	*CDC7*	0.4	0.41	*CDC25A*	0.42	0.39	*TTK*	0.23	0.3
*TGFB1*	−0.24	−0.34	*TIMELESS*	0.55	0.51	*TIMELESS*	0.55	0.51	*BUB1*	0.28	0.33
*PPM1G*	0.31	0.25							*FBXO5*	0.29	0.3
*KHDRBS1*	0.33	0.28							*NEK2*	0.29	0.3
*CDK2*	0.34	0.43							*CHFR*	0.3	0.21
*HCFC1*	0.39	0.39							*CDC25C*	0.31	0.34
*CDC25A*	0.42	0.39							*ZWINT*	0.33	0.35
									*BUB1B*	0.34	0.37
									*NUSAP1*	0.36	0.38
									*KNTC1*	0.62	0.57

Correlations were calculated between expression of a curated list of cell cycle regulatory genes and KDM2B. The genes listed here had Pearson or Spearman coefficients of ⩾0.3 with *P*<0.0001. The correlated genes are separated by phase of the cell cycle regulated. All genes in the list also correlate significantly with EZH2 expression except NUSAP1 (*P*=0.072).
